# Supplementary material for: Blood donations and donors’ profile in Lithuania: Trends for coming back after the COVID-19 outbreak
Source: PLoS One. 2024 Jan 25;19(1):e0297580. doi: 10.1371/journal.pone.0297580 (PMC10810517; doi:10.1371/journal.pone.0297580)
Supplement: S1 Table — (DOCX) [file pone.0297580.s001.docx]

**S1 Table. The score values of donations proportions’ comparisons according to specific dimensions in Lithuania between April 2019 and March 2023**

| Dimensions of donations | Pre-pandemic year compared  with | | | 1-st  pandemic year compared  with | | 2-nd pandemic year compared with  3-rd  pandemic year |
| --- | --- | --- | --- | --- | --- | --- |
|  | 1-st  pandemic year | 2-nd pandemic year | 3-rd  pandemic year | 2-nd pandemic year | 3-rd  pandemic year |  |
| *Type of remuneration* | | | | | | |
| Voluntary, non –remunerated | z=17.78  **P<0.001** | z=18.86  **P<0.001** | z=19.35  **P<0.001** | z=2.08  **P<0.05** | z=2.14  **P<0.05** | N/A |
| Remunerated | z=19.95  **P<0.001** | z=21.09  **P<0.001** | z=21.963  **P<0.001** | z=2.08  **P<0.05** | z=2.14  **P<0.05** | N/A |
| *Type of blood donor* | | | | | | |
| First-time | z=40.86  **P<0.001** | z=36.39  **P<0.001** | z=35.29  **P<0.001** | z=5.49  **P<0.001** | z=7.16  **P<0.001** | z=1.64  P>0.05 |
| Repeat/regular | z=40.86  **P<0.001** | z=36.22  **P<0.001** | z=35.29  **P<0.001** | z=5.66  **P<0.001** | z=7.16  **P<0.001** | z=1.47  P>0.05 |
| *Place of donation* | | | | | | |
| Blood establishment | z=73.32  **P<0.001** | z=56.35  **P<0.001** | z=44.79  **P<0.001** | z=18.18  **P<0.001** | z=30.98  **P<0.001** | z=12.27  **P<0.001** |
| Mobile session | z=73.31  **P<0.001** | z=56.35  **P<0.001** | z=44.79  **P<0.001** | z=18.18  **P<0.001** | z=30.39  **P<0.001** | z=12.27  **P<0.001** |
| *Type of donation* | | | | | | |
| Whole blood | z=14.34  **P<0.001** | z=0.33  P>0.05 | z=3.349  P>0.05 | z=13.95  **P<0.001** | z=17.88  **P<0.001** | z=3.381  **P<0.001** |
| Double RBC apheresis | z=8.84  **P<0.001** | z=2.59  **P<0.05** | z=6.67  **P<0.001** | z=11.33  **P<0.001** | z=15.42  **P<0.001** | z=4.03  **P<0.001** |
| RBC apheresis | z=2.58  **P<0.05** | z=4.06  **P<0.001** | z=4.90  **P<0.001** | z=1.59  P>0.05 | z=2.59  **P<0.05** | z=1.08  P>0.05 |
| Plasmapheresis | z=29.08  **P<0.001** | z=15.376  **P<0.001** | z=14.91  **P<0.001** | z=17.68  **P<0.001** | z=19.05  **P<0.001** | z=1.19  P>0.05 |
| Platelet apheresis | z=11.75  **P<0.001** | z=0.83  P>0.05 | z=4.43  **P<0.001** | z=10.92  **P<0.001** | z=7.59  **P<0.001** | z=3.057  **P<0.05** |
| Plasma and platelet apheresis | z=26.85  **P<0.001** | z=3.41  **P<0.05** | z=10.70  **P<0.001** | z=24.31  **P<0.001** | z=18.47  **P<0.001** | z=7.49  **P<0.001** |
