# Supplementary material for: Blood donations and donors’ profile in Lithuania: Trends for coming back after the COVID-19 outbreak
Source: PLoS One. 2024 Jan 25;19(1):e0297580. doi: 10.1371/journal.pone.0297580 (PMC10810517; doi:10.1371/journal.pone.0297580)
Supplement: S2 Table — (DOCX) [file pone.0297580.s002.docx]

**S2 Table. The score values of donations proportions’ comparisons according to donor’s age and sex in different years in Lithuania between April 2019 and March 2023**

| Blood and its components donors | Pre-pandemic year compared  with | | | 1-st  pandemic year copmared  with | | 2-nd pandemic year compared with  3-rd  pandemic year |
| --- | --- | --- | --- | --- | --- | --- |
|  | 1-st  pandemic year | 2-nd  pandemic year | 3-rd  pandemic year | 2-nd  pandemic year | 3-rd  pandemic year |  |
| *Males* | | | | | | |
| Under 25 years old | z=19.79  **P<0.001** | z=20.51  **P<0.001** | z=31.19  **P<0.001** | z=0.24  P>0.05 | z=10.48  **P<0.001** | z=10.44  **P<0.001** |
| 25-34 years old | z=4.59  **P<0.001** | z=3.24  **P<0.05** | z=1.72  P>0.05 | z=1.42  P>0.05 | z=2.96  **P<0.05** | z=1.55  P>0.05 |
| 35-44 years old | z=11.38  **P<0.001** | z=14.03  **P<0.001** | z=20.43  **P<0.001** | z=2.36  **P<0.05** | z=8.47  **P<0.001** | z=6.22  **P<0.001** |
| 45-54 years old | z=8.81  **P<0.001** | z=15.92  **P<0.001** | z=23.36  **P<0.001** | z=6.77  **P<0.001** | z=13.93  **P<0.001** | z=7.28  **P<0.001** |
| 55-64 years old | z=4.34  **P<0.001** | z=9.35  **P<0.001** | z=15.40  **P<0.001** | z=4.79  **P<0.001** | z=10.65  **P<0.001** | z=5.98  **P<0.001** |
| 65 and over years old | z=0.47  P>0.05 | z=1.76  P>0.05 | z=2.02  **P<0.05** | z=2.16  **P<0.05** | z=2.42  **P<0.05** | z=0.25  P>0.05 |
| All males | z=6.17  **P<0.001** | z=13.95  **P<0.001** | z=17.85  **P<0.001** | z=7.46  **P<0.001** | z=11.18  **P<0.001** | z=3.71  **P<0.05** |
| *Females* | | | | | | |
| Under 25 years old | z=16.86  **P<0.001** | z=30.35  **P<0.001** | z=39.51  **P<0.001** | z=12.93  **P<0.001** | z=21.77  **P<0.001** | z=8.86  **P<0.001** |
| 25-34 years old | z=1.41  P>0.05 | z=8.06  **P<0.001** | z=13.43  **P<0.001** | z=9.28  **P<0.001** | z=14.54  **P<0.001** | z=5.24  **P<0.001** |
| 35-44 years old | z=2.34  **P<0.05** | z=1.04  P>0.05 | z=2.18  **P<0.05** | z=1.32  P>0.05 | z=0.23  P>0.05 | z=1.13  P>0.05 |
| 45-54 years old | z=3.19  **P<0.05** | z=8.61  **P<0.001** | z=12.50  **P<0.001** | z=5.22  **P<0.001** | z=8.96  **P<0.001** | z=3.78  **P<0.05** |
| 55-64 years old | z=0.83  P>0.05 | z=7.61  **P<0.001** | z=9.80  **P<0.001** | z=8.23  **P<0.001** | z=10.35  **P<0.001** | z=2.10  **P<0.05** |
| 65 and over years old | z=1.91  P>0.05 | z=2.49  **P<0.05** | z=4.10  **P<0.001** | z=4.15  **P<0.001** | z=5.56  **P<0.001** | z=1.66  P>0.05 |
| All females | z=6.17  **P<0.001** | z=13.95  **P<0.001** | z=17.85  **P<0.001** | z=7.46  **P<0.001** | z=11.18  **P<0.001** | z=3.71  **P<0.05** |
| *Both males and females* | | | | | | |
| Under 25 years old | z=27.58  **P<0.001** | z=37.58  **P<0.001** | z=52.26  **P<0.001** | z=9.20  **P<0.001** | z=23.31  **P<0.001** | z=14.27  **P<0.001** |
| 25-34 years old | z=4.87  **P<0.001** | z=2.67  **P<0.05** | z=7.49  **P<0.001** | z=7.46  **P<0.001** | z=12.22  **P<0.001** | z=4.77  **P<0.001** |
| 35-44 years old | z=11.06  **P<0.001** | z=12.37  **P<0.001** | z=18.59  **P<0.001** | z=1.06  P>0.05 | z=6.98  **P<0.001** | z=6.05  **P<0.001** |
| 45-54 years old | z=9.10  **P<0.001** | z=18.52  **P<0.001** | z=27.17  **P<0.001** | z=9.00  **P<0.001** | z=17.34  **P<0.001** | z=8.44  **P<0.001** |
| 55-64 years old | z=2.45  **P<0.05** | z=13.88  **P<0.001** | z=18.06  **P<0.001** | z=11.08  **P<0.001** | z=15.11  **P<0.001** | z=4.04  **P<0.001** |
| 65 and over years old | z=1.45  P>0.05 | z=2.94  **P<0.05** | z=4.25  **P<0.001** | z=4.23  **P<0.001** | z=5.46  **P<0.001** | z=1.31  P>0.05 |
