# Supplementary material for: Blood donations and donors’ profile in Lithuania: Trends for coming back after the COVID-19 outbreak
Source: PLoS One. 2024 Jan 25;19(1):e0297580. doi: 10.1371/journal.pone.0297580 (PMC10810517; doi:10.1371/journal.pone.0297580)
Supplement: S3 Table — (DOCX) [file pone.0297580.s003.docx]

**S3 Table. The score values of proportions’ comparisons of blood and its components donors’ deferrals in Lithuania between April 2019 and March 2023**

| Dimensions | Pre-pandemic year  compared with | | | 1-st pandemic year  compared with | | 2-nd pandemic year compared with  3-rd  pandemic year |
| --- | --- | --- | --- | --- | --- | --- |
|  | 1-st  pandemic year | 2-nd  pandemic year | 3-rd  pandemic year | 2-nd  pandemic year | 3-rd  pandemic year |  |
| Pre-donation deferrals | z=16.59  **P<0.001** | z=8.55  **P<0.001** | z=11.96  **P<0.001** | z=8.24  **P<0.001** | z=5.12  **P<0.001** | z=3.29  **P<0.05** |
| *Permanent deferrals* | | | | | | |
| Positive TTI markers | z=0.54  P>0.05 | z=1.70  P>0.05 | z=3.63  **P<0.05** | z=1.01  P>0.05 | z=2.71  **P<0.05** | z=1.78  P>0.05 |
| Somatic illnesses or conditions | z=1.21  P>0.05 | z=0.51  P>0.05 | z=1.54  P>0.05 | z=1.60  P>0.05 | z=2.53  **P<0.05** | z=0.96  P>0.05 |
| Infectious diseases | z=0.07  P>0.05 | z=2.17  **P<0.05** | z=0.07  P>0.05 | z=2.22  **P<0.05** | z=1.243  P>0.05 | z=3.32  **P<0.05** |
| Drug usage | z=0.20  P>0.05 | z=1.14  P>0.05 | z=0.62  P>0.05 | z=1.13  P>0.05 | z=0.71  P>0.05 | z=0.52  P>0.05 |
| Other reasons | z=3.19  **P<0.05** | z=2.67  **P<0.05** | z=2.88  **P<0.05** | z=0.59  P>0.05 | z=0.46  P>0.05 | z=0.15  P>0.05 |
| *Temporary deferrals* | | | | | | |
| Inappropriate level of hemoglobin | z=7.72  **P<0.001** | z=13.67  **P<0.001** | z=1.71  P>0.05 | z=5.14  **P<0.001** | z=5.97  **P<0.001** | z=11.367  **P<0.001** |
| Inappropriate blood pressure, heart rate, or rhytm | z=6.35  **P<0.001** | z=0.23  P>0.05 | z=0.76  P>0.05 | z=5.96  **P<0.001** | z=5.49  **P<0.001** | z=0.51  P>0.05 |
| Surgery operation and/or intervention | z=2.51  **P<0.05** | z=5.20  **P<0.001** | z=8.10  **P<0.001** | z=7.17  **P<0.001** | z=9.75  **P<0.001** | z=2.81  **P<0.05** |
| Medication usage | z=2.62  **P<0.05** | z=3.15  **P<0.05** | z=3.66  **P<0.001** | z=0.35  P>0.05 | z=5.89  **P<0.001** | z=6.61  **P<0.001** |
| Tattoos and/or body piercing | z=4.35  **P<0.001** | z=1.49  P>0.05 | z=0.48  P>0.05 | z=2.89  **P<0.05** | z=3.83  **P<0.001** | z=0.99  P>0.05 |
| Refusal to donate | z=0.85  P>0.05 | z=5.27  **P<0.001** | z=2.02  **P<0.05** | z=4.17  **P<0.001** | z=1.04  P>0.05 | z=3.26  **P<0.05** |
| Inappropriate body weight and/or age | z=2.47  **P<0.05** | z=2.39  **P<0.05** | z=4.29  **P<0.001** | z=0.22  P>0.05 | z=1.61  P>0.05 | z=1.91  P>0.05 |
| Other reasons | z=9.49  **P<0.001** | z=16.89  **P<0.001** | z=7.23  **P<0.001** | z=6.48  **P<0.001** | z=2.63  **P<0.05** | z=9.55  **P<0.001** |
