# Supplementary material for: Blood donations and donors’ profile in Lithuania: Trends for coming back after the COVID-19 outbreak
Source: PLoS One. 2024 Jan 25;19(1):e0297580. doi: 10.1371/journal.pone.0297580 (PMC10810517; doi:10.1371/journal.pone.0297580)
Supplement: S4 Table — (DOCX) [file pone.0297580.s004.docx]

**S4 Table. The score values of prevalence comparisons of transfusion-transmitted infectious disease markers in blood and its components’ donations in Lithuania between April 2019 and March 2023**

| Confirmed TTI markers | Pre-pandemic year compared  with | | | 1-st  pandemic year compared  with | | 2-nd pandemic year compared with  3-rd  pandemic year |
| --- | --- | --- | --- | --- | --- | --- |
|  | 1-st  pandemic year | 2-nd  pandemic year | 3-rd  pandemic year | 2-nd  pandemic year | 3-rd  pandemic year |  |
| *First-time donations* | | | | | | |
| HBV | z=1.29  P>0.05 | z=1.46  P>0.05 | z=3.01  **P<0.05** | z=0.11  P>0.05 | z=1.52  P>0.05 | z=1.46  P>0.05 |
| HCV | z=1.00  P>0.05 | z=1.49  P>0.05 | z=0.93  P>0.05 | z=0.39  P>0.05 | z=0.14  P>0.05 | z=0.56  P>0.05 |
| Syphilis | z=0.25  P>0.05 | z=0.31  P>0.05 | z=0.69  P>0.05 | z=0.50  P>0.05 | z=0.82  P>0.05 | z=0.33  P>0.05 |
| HIV 1 and 2 | z=1.06  P>0.05 | z=0.43  P>0.05 | z=0.27  P>0.05 | z=1.31  P>0.05 | z=0.89  P>0.05 | z=0.64  P>0.05 |
| All TTI markers  among first-time donations | z=1.65  P>0.05 | z=1.61  P>0.05 | z=2.14  **P<0.05** | z=0.12  P>0.05 | z=0.32  P>0.05 | z=0.45  P>0.05 |
| *Repeat/regular donations* | | | | | | |
| HBV | z=1.22  P>0.05 | z=0.36  P>0.05 | z=0.45  P>0.05 | z=0.89  P>0.05 | z=0.82  P>0.05 | z=0.08  P>0.05 |
| HCV | z=0.31  P>0.05 | z=0.66  P>0.05 | z=0.18  P>0.05 | z=0.97  P>0.05 | z=0.50  P>0.05 | z=0.49  P>0.05 |
| Syphilis | z=1.02  P>0.05 | z=0.34  P>0.05 | z=0.69  P>0.05 | z=1.35  P>0.05 | z=0.37  P>0.05 | z=1.04  P>0.05 |
| HIV 1 and 2 | z=0.75  P>0.05 | z=0.50  P>0.05 | z=0.57  P>0.05 | z=1.23  P>0.05 | z=0.19  P>0.05 | z=1.07  P>0.05 |
| All TTI markers  among repeat/regular  donations | z=0.63  P>0.05 | z=0.45  P>0.05 | z=0.48  P>0.05 | z=0.20  P>0.05 | z=0.18  P>0.05 | z=0.03  P>0.05 |
| All TTI markers  among all (first-time  and repeat/regular)  donations | z=4.79  **P<0.001** | z=4.36  **P<0.001** | z=4.72  **P<0.001** | z=0.57  P>0.05 | z=0.29  P>0.05 | z=0.29  P>0.05 |
