# Supplementary material for: Blood donations and donors’ profile in Lithuania: Trends for coming back after the COVID-19 outbreak
Source: PLoS One. 2024 Jan 25;19(1):e0297580. doi: 10.1371/journal.pone.0297580 (PMC10810517; doi:10.1371/journal.pone.0297580)
Supplement: Attachment — Submitted filename: Response to Reviewers.docx [file pone.0297580.s005.docx]

Editors

PLOS ONE

October 5, 2023

Dear Editor,

We thank you and the reviewers for a thorough reading and constructive suggestions to improve our manuscript and for the opportunity to revise and resubmit it. We are pleased to submit the improved research article “Blood donations and donors' profile in Lithuania: trends for coming back after the COVID-19 outbreak?“ for your consideration for publication in PLOS ONE. Below you will find our response to the Editor comments.

On behalf of the authors, I thank you for your consideration of this resubmission. We appreciate your time and look forward to your response.

Sincerely,

Prof. Vytenis Kalibatas, MD, MPH, PhD (corresponding author)

Department of Health Management,

Lithuanian University of Health Sciences

Tilžės str. 18, LT-47181, Kaunas, Lithuania

E-mail: vytenis.kalibatas@lsmuni.lt

Journal Requirements

*When submitting your revision, we need you to address these additional requirements.*

*1. Please ensure that your manuscript meets PLOS ONE's style requirements, including those for file naming. The PLOS ONE style templates can be found at* 
[*https://journals.plos.org/plosone/s/file?id=wjVg/PLOSOne_formatting_sample_main_body.pdf*](https://journals.plos.org/plosone/s/file?id=wjVg/PLOSOne_formatting_sample_main_body.pdf)  *and* 
[*https://journals.plos.org/plosone/s/file?id=ba62/PLOSOne_formatting_sample_title_authors_affiliations.pdf*](https://journals.plos.org/plosone/s/file?id=ba62/PLOSOne_formatting_sample_title_authors_affiliations.pdf)

We thank you for the advices on the revision. We followed the templates and changed the first page and level 2 headings in “Material and Methods” section of the manuscript according to the requirements.

*2. Please amend either the title on the online submission form (via Edit Submission) or the title in the manuscript so that they are identical.*

We changed the title on the online submission form.

3. *Please review your reference list to ensure that it is complete and correct. If you have cited papers that have been retracted, please include the rationale for doing so in the manuscript text, or remove these references and replace them with relevant current references. Any changes to the reference list should be mentioned in the rebuttal letter that accompanies your revised manuscript. If you need to cite a retracted article, indicate the article’s retracted status in the References list and also include a citation and full reference for the retraction notice.*

We reviewed the reference list, and it is complete and correct. To the best of our knowledge, we did not cite any retracted article.

We have cited only one document, which has been canceled, i.e. Decision of the Government of the Republic of Lithuania on 14-03-2020 № 270, declaring a quarantine in the territory of the Republic of Lithuania (Reference № 13). The rationale for citing this reference is explained in the manuscript Material and Methods section: due to the particular period of declaring the quarantine, we determined that the one-year study period would start on April 1 and end on March 31. We added information that this Decision was later canceled, and provided a new reference to the document canceling this legal act.

4. Responses to Additional Editor Comments:

| **№** | **Comments** | **Responses** |
| --- | --- | --- |
| 1. | Remove the Objective subheading on page 12 of the PDF document. Merge the content under the objective with the introduction | Done.  The Objective subheading is removed, and content is merged with the Introduction section. |
| 2. | Write the Materials and Methods section in themes | Done.  Materials and Methods section is divided into subsections: Data collection, Study period, Donor screening and deferral, Statistical analysis and Ethical considerations. |
