# Supplementary material for: Blood donations and donors’ profile in Lithuania: Trends for coming back after the COVID-19 outbreak
Source: PLoS One. 2024 Jan 25;19(1):e0297580. doi: 10.1371/journal.pone.0297580 (PMC10810517; doi:10.1371/journal.pone.0297580)
Supplement: Attachment — Submitted filename: Response to Reviewers.docx [file pone.0297580.s006.docx]

Editors

PLOS ONE

October 11, 2023

Dear Editor,

We thank you and the reviewers for a thorough reading and for the opportunity to resubmit it. We are pleased to submit the improved research article “Blood donations and donors' profile in Lithuania: trends for coming back after the COVID-19 outbreak?“ for your consideration for publication in PLOS ONE.

Response to Academic Editor:

Upload a clean version of the manuscript – The new version of the manuscript is uploaded.

On behalf of the authors, I thank you for your consideration of this resubmission. We appreciate your time and look forward to your response.

Sincerely,

Prof. Vytenis Kalibatas, MD, MPH, PhD (corresponding author)

Department of Health Management,

Lithuanian University of Health Sciences

Tilžės str. 18, LT-47181, Kaunas, Lithuania

E-mail: vytenis.kalibatas@lsmuni.lt
