# Supplementary material for: Blood donations and donors’ profile in Lithuania: Trends for coming back after the COVID-19 outbreak
Source: PLoS One. 2024 Jan 25;19(1):e0297580. doi: 10.1371/journal.pone.0297580 (PMC10810517; doi:10.1371/journal.pone.0297580)
Supplement: Attachment — Submitted filename: Manuscript PONE D 23 REVIEWED.docx [file pone.0297580.s008.docx]

**Blood donations and donors' profile in Lithuania: trends for coming back after the COVID-19 outbreak?**

Vytenis Kalibatas^1*¶^, Lina Kalibatienė^2¶^, Dulat Imashpayev^3¶^

**^1^** Department of Health Management, Lithuanian University of Health Sciences, Kaunas, Lithuania

^2^ Department of Anaesthesiogy, Lithuanian University of Health Sciences, Kaunas, Lithuania

^3^ Scientific and Production Center of Transfusiology, Astana, Kazakhstan

*Corresponding Author

E-mail: [vytenis.kalibatas@lsmu.lt](mailto:vytenis.kalibatas@lsmu.lt) (VK)

^¶^The authors contributed equally to this work

**Abstract**

The coronavirus disease (COVID-19) pandemic has significantly affected blood donors worldwide. It is important for the blood service to return to its pre-pandemic level as soon as possible and to perform its functions fully. This study compared the donation and demographic profiles of blood and its component donors one year before and during three pandemic years in Lithuania. All blood and blood component donations (n=413,358) and demographic characteristics of all donors from April 1, 2019, to March 31, 2023, were analyzed. All data were obtained from annual publications, and statistics were obtained from the Blood Donor Register. Data were analyzed using descriptive statistics. Following a 9.41 percent decrease in the first year of the pandemic, the quantity of blood and blood component donations increased by 3.49 percent in the third year compared to the pre-pandemic year. Throughout the three years of the pandemic, a statistically significant decrease in the proportion of first-time blood and blood component donations was observed. Both the number and proportion of donations by donors under 25 decreased during the pandemic. The proportion of pre-donation deferrals for all attempts to donate significantly decreased during the pandemic. There was a statistically significant lower prevalence of all positive transfusion-transmitted infectious (TTI) markers among all donations compared to the pre-pandemic year for all three pandemic years. The odds for HBV, HCV, and all TTI markers during the second and third pandemic years were significantly lower than those in the pre-pandemic year. In conclusion, most dimensions of blood and its component donations and donor characteristics have returned to pre-pandemic levels or show positive trends. However, the major concern is the remaining decrease in donations from first-time and donors under 25.

**Key words:** COVID-19, blood donation, blood donors, deferral, transfusion-transmitted infections

**Introduction**

The first cases of novel coronavirus were first detected in China in December 2019, with the virus spreading rapidly to other countries worldwide. This led the WHO to declare a Public Health Emergency of International Concern (PHEIC) on January 30, 2020, and to characterize the outbreak as a pandemic on March 11, 2020. On May 5, 2023, the WHO Emergency Committee on COVID-19 recommended recognizing that, given that the disease was well-established and ongoing, it no longer fit the definition of a PHEIC[1]. Many aspects of human life have been disrupted by the COVID-19 pandemic[2], which had massive consequences on societies and health systems across countries[3].

Blood transfusions are a vital component of every country's health service[4]. Ensuring a sufficient and safe supply of blood and blood products is vital for the effective functioning of healthcare systems and patients' overall well-being. However, ensuring a sufficient supply of safe blood may prove challenging because disasters or other emergencies may damage the available civil and healthcare infrastructure, disrupting mobility, transportation, and service provision. Moreover, some population members may not be able to donate blood because of fear or illness[5]. The COVID-19 pandemic containment measures imposed to mitigate the spread of COVID-19, including lockdown, social distancing guidelines, and travel restrictions, have disrupted regular blood donation processes. This has caused a significant decrease in the number of blood donations worldwide and has become a serious issue [6]. A few scientific studies have revealed that the profile of donors changed during the pandemic period: the COVID-19 pandemic impacted the type (first time/repeat or regular) of blood donations and demographic profile (age and sex) of blood donors [7,8]. Other studies have analyzed blood safety in terms of the prevalence of infectious disease markers [9,10] and donor deferrals to donate blood and its components during the COVID-19 pandemic [11].

Although considerable publications are focusing on the various consequences of the COVID-19 pandemic on blood transfusion services, there are no publications on the impact of the COVID-19 pandemic on blood donations and donor profiles over an extended period, revealing important trends after drastic changes that occurred during the initial stages of the pandemic.

This study aimed to assess the impact of the COVID-19 pandemic on the number, type, and safety of blood donations and the demographic profile of blood donors and its components, comparing the years before and during the pandemic and examining trends over three pandemic years.

**Materials and Methods**

**Data collection**

All blood and blood component donations and the demographic characteristics of all donors in Lithuania from April 1, 2019, to March 31, 2023, were analyzed. All data were obtained from statistical reports of the National Blood Donors´ register[12]. During this study, the following dimensions were analyzed: the total number of blood and its components donated; number of donations according to donation type (first-time/regular or repeat); donors’ demographic characteristics (age and sex); procedure type (whole blood, red blood cell apheresis, platelet apheresis, plasmapheresis); the prevalence of HIV 1 and 2, HBV, HCV, and syphilis markers per 100 donations; the causes of donation restrictions; and temporary deferrals.

**Study period**

The study period was 4 years. Like most countries, Lithuania declared quarantine in mid-March 2021 and restricted regular human and institutional activities [13] (which was later canceled [14]). Thus, we determined that the one-year study period would start on April 1 and end on March 31. The period from April 1, 2019, to March 31, 2020, was considered the pre-pandemic year; the period from April 1, 2020, to March 31, 2021, was considered the first year of the pandemic; the period from April 1, 2021, to March 31, 2022, was considered the second year of the pandemic; and the period from April 1, 2022, to March 31, 2023, was considered the third year of the pandemic.

**Donor screening and deferral**

Blood and blood components were tested for serological anti-HCV, HBV surface antigen (HBsAg), Ag/anti-HIV 1 and 2, and syphilis antibodies. If the results of serological screening were positive or uncertain, the individual sample test was repeated. If any of the repeated tests yielded positive or uncertain results, the subsequent confirmatory tests employed were as follows: the HBsAg neutralization test for HBV, the anti-HCV immunoblot test for HCV, Western blotting and/or HIV 1–p24 antigen and/or HIV 2 ELISA for HIV 1 and 2, and the Treponema Pallidum hemagglutination (TPHA) test for syphilis. Seronegative donors were tested for HCV, HBV, and HIV-1 infections using a nucleic acid test (NAT). If the results of individual samples were reactive for HIV RNA, HCV RNA, or HBV DNA, a confirmatory quantitative test was performed [15]. Donations with confirmed infectious disease markers (serological or NAT) were considered positive.

The criteria for permanent donation restriction and temporary deferrals of blood and its components were set up by the Ministry of Health of the Republic of Lithuania [16]. Donors of blood or its components may be permanently restricted from donating for one or more of the following reasons: circulatory system disease, disease of the central nervous system, increased risk of bleeding, repeated fainting cases or former seizures, diabetes, malignant diseases, use of intravenous drugs and/or oral/intramuscular anabolic steroids or hormonal substances, and some infectious and other diseases. Temporary deferrals involve the following reasons: the current physiological and health status of the donor (age under 18 years, weight, inappropriate hemoglobin level, blood pressure or heart rate, fever, rheumatism, some infectious and other diseases), recent surgical operations or procedures, tattooing and ring piercing, vaccination, pregnancy, and the use of some medicines. Deferral periods vary for various reasons.

**Statistical analysis**

The chi-squared test was used to compare multiple proportions, whereas the z-test was used to compare two proportions. Prevalence was calculated as the ratio of the number of confirmed positive results for transfusion-transmitted infectious (TTI) disease markers (serological syphilis, anti-HCV, HBsAg, anti-HIV 1 and 2, and/or HCV, HBV, and HIV-1 NAT) per 100 donations. Odds ratios (OR) and 95% confidence intervals (CIs) were determined. Statistical significance was set at P <0.05. Data analysis was conducted using SPSS software (version 29,0).

**Ethical considerations**

As per local legislation, this survey is not the subject of bioethical regulation because generalized, fully anonymized, and publicly available data (as opposed to collecting personal data directly from research participants, medical health records, or archived samples) have been used. The Kaunas Regional Biomedical Research Ethics Committee reviewed the methodology of this survey and deemed the investigation an evaluation of service, not requiring review by an ethics committee, as the direct object of this study was not a specific person or person´s health. The analysis of de-identified, fully anonymized, generalized, and publicly available data did not constitute human subject research and did not require the participants’ consent.

**Results**

A cumulative count of 105,321 blood and component donations was recorded during the pre-pandemic period in Lithuania. During the first year of the COVID-19 pandemic, blood and blood component donations decreased by 9.41 percent, and during the second year, they decreased by 1.60 percent compared to pre-pandemic years. In the third year of the pandemic, the number of blood and blood component donations increased by 3.49 percent compared with the pre-pandemic year (Table 1).

In the pre-pandemic year, a total of 361 blood and its component donations were compensated (constituting 0.34 percent of all donations). In the second year, this number dropped significantly to four donations (0.004 percent of all donations) in Lithuania. Notably, no remunerated donations were recorded during the second and third years of the pandemic.

Throughout the three years of the COVID-19 pandemic, a statistically significant decrease in the proportion of first-time blood and blood component donations was observed. The proportion of first-time donations was significantly lower during the first (z=40.86; P<0.001), second (z=36.39, P<0.001), and third (z=35.3, P<0.001) pandemic years compared to the pre-pandemic year and did not reach the pre-pandemic level. There was also a decrease in blood and blood component donations during mobile blood-driving. The proportion of donated blood during mobile blood drives was significantly lower during the first (z=73.32, P<0.001), second (z=56.36, P<0.001), and third pandemic years (z=44.8, P<0.001) than during the pre-pandemic year. Despite this positive trend, neither the number nor the proportion of donations in mobile sessions reached pre-pandemic levels.

**Table 1. Number of blood donations according to specific dimensions in Lithuania between April 2019 and March 2023**

| Dimensions of donations | Years | | | | % change (+/-), comparing  Pre-pandemic year with | | |
| --- | --- | --- | --- | --- | --- | --- | --- |
|  | Pre-pandemic  No (%*) | 1^st^ pandemic  No (%*) | 2^nd^ pandemic  No (%*) | 3^rd^ pandemic  No (%*) | 1^st^ pandemic | 2^nd^  pandemic | 3^rd^  pandemic |
| Total blood and components donations | 105321 | 95409 | 103631 | 108997 | -9.41 | -1.60 | +3.49 |
| *Type of blood donor* | | | | | | | |
| First-time | 16820 (15.97) | 9368 (9.82) | 10949 (10.57) | 11756 (10.79) | -44.30 | -34.90 | -30.11 |
| Repeat/regular | 88501 (84.03) | 86041 (90.18) | 92658 (89.43) | 97241 (89.21 | -2.78 | +4.70 | +9.86 |
| *Place of donation* | | | | | | | |
| Blood establishment | 34787 (33.03) | 46871 (49.13) | 46692 (45.06) | 46231 (42.41) | +34.74 | +34.22 | +32.89 |
| Mobile session | 70534 (66.97) | 48538 (50.87) | 56939 (54.94) | 62766 (57.59) | -31.18 | -19.27 | -11.01 |
| *Type of donation* | | | | | | | |
| Whole blood | 99664 (94.63) | 88818 (93.09) | 98031 (94.60) | 103508 (94.96) | -10.88 | -1.64 | +3.86 |
| Double RBC apheresis | 2764 (2.62) | 3141 (3.29) | 2535 (2.45) | 2380 (2.18) | +13.64 | -8.29 | -13.90 |
| RBC apheresis | 31 (0.029) | 12 (0.01) | 6 (0.01) | 3 (0,003) | -61.29 | -80.65 | -90.32 |
| Plasmapheresis | 1 (0.001) | 766 (0.80) | 247 (0.24) | 233 (0.217) | +>100 | +>100 | +>100 |
| Platelet apheresis | 2747 (2.61) | 1747 (1.83) | 2643 (2.55) | 2520 (2.31) | -36.40 | -3.79 | -8.26 |
| Plasma and platelet apheresis | 114 (0.11) | 925 (0.97) | 169 (0.16) | 353 (0.32) | +>100 | +48.25 | +>100 |

*From total donations during particular year

Compared to the pre-pandemic years, statistically significant differences were observed in the proportion of donations based on the item being donated. The proportion of whole blood donations to the total number of donations was significantly lower than that in the pre-pandemic year (z=14.34, P<0.001). Moreover, the proportion of platelet apheresis donations was significantly lower during the first year of the pandemic than during the pre-pandemic year (z=11.8, P<0.001). During the third pandemic year, the proportion of donations based on the item donated was very similar to that in the pre-pandemic year, except for an increase in plasmapheresis and plasma and platelet apheresis donations and a decrease in red blood cell apheresis.

There were statistically significant differences in donor age and sex between the pre-pandemic and pandemic years. Both the number and proportion of donors aged <25 years decreased during the pandemic (Table 2). The proportions of donations of males and females under 25 during the pre-pandemic year were significantly higher than during the pandemic years (z=27.58, P<0.001; z=37.58, P<0.001; and z=52.26, P<0.001, respectively). The proportions of donations of males and females 35–44 years old, 45–55 years old, 55–64 years old, and 65 and older were statistically significantly higher compared with pre-pandemic years. The proportion of male donors showed a tendency to increase during the pandemic year, with statistically significant differences compared with the pre-pandemic year (z=6.16, P<0.001; z=13.97, P<0.001; and z=17.84, P<0.001, respectively).

**Table 2. Number of donations according to donors’ age and sex**

| Blood and its components donors | Years | | | | % change (+/-), comparing  Pre-pandemic year with | | |
| --- | --- | --- | --- | --- | --- | --- | --- |
|  | Pre-pandemic  No (%*) | 1^st^ pandemic  No (%*) | 2^nd^ pandemic  No (%*) | 3^rd^ pandemic  No (%*) | 1^st^ pandemic | 2^nd^  pandemic | 3^rd^  pandemic |
| *Males* | | | | | | | |
| Under 25 years old | 13047 (12.39) | 9172 (9.61) | 9929 (9.58) | 9036 (8.29) | -29.70 | -23.90 | -30.74 |
| 25–34 years old | 18842 (17.89) | 17826 (18.68) | 19106 (18.44) | 19811 (18.17) | -5.39 | +1.40 | +5.14 |
| 35–44 years old | 16097 (15.28) | 16369 (17.16) | 18195 (17.56) | 20270 (18.60) | +1.69 | +13.03 | +25.92 |
| 45–54 years old | 9500 (9.02) | 9711 (10.18) | 11519 (11.11) | 13219 (12.13) | +2.22 | +21.25 | +39.15 |
| 55–64 years old | 2697 (2.56) | 2744 (2.88) | 3565 (3.44) | 4058 (3.72) | +1.74 | +32.18 | +50.46 |
| 65 and over years old | 43 (0.04) | 35 (0.03) | 60 (0.06) | 66 (0.06) | -18.60 | +39.53 | +53.49 |
| In total | 60226 (57.18) | 55857 (58.54) | 62374 (60.19) | 66460 (60.97) | -7.25 | +3.57 | +10.35 |
| *Females* | | | | | | | |
| Under 25 years old | 10952 (10.40) | 7827 (8.20) | 6927 (6.69) | 6275 (5.76) | -28.53 | -36.75 | -42.70 |
| 25–34 years old | 11445 (10.87) | 10556 (11.07) | 10149 (9.79) | 9949 (9.13) | -7.77 | -11.32 | -13.07 |
| 35–44 years old | 11531 (10.95) | 10759 (11.28) | 11493 (11.09) | 12256 (11.24) | -6.69 | -0.33 | +6.29 |
| 45–54 years old | 8017 (7.61) | 7627 (7.99) | 8955 (8.64) | 9927 (9.11) | -4.86 | +11.70 | +23.82 |
| 55–64 years old | 3126 (2.97) | 2772 (2.91) | 3689 (3.56) | 4066 (3.73) | -11.32 | +18.01 | +30.07 |
| 65 and over years old | 24 (0.02) | 11 (0.01) | 44 (0.04) | 64 (0.06) | -54.17 | +83.33 | +>100 |
| In total | 45095 (42.82) | 39552 (41.46) | 41257 (39.81) | 42537 (39.03) | -12.29 | -8.51 | -5.67 |

*From total donations during particular year

During the pre-pandemic year, there were 122509 attempts to donate blood and its components and 17188 pre-donation deferrals (deferral rate, 14.03). During the pandemic, the number of pre-donation deferrals decreased (Table 3). The proportions of pre-donation deferrals of all attempts to donate significantly decreased during the pandemic years (z=16.44, P<0.001; z=8.94, P<0.001; and z=12.7, P<0.001, respectively) compared with pre-pandemic years.

During the pre-pandemic year, the main reasons for permanent donor deferral were positive TTI disease markers and somatic illnesses. The proportion of positive TTI markers for all deferrals significantly decreased during the third pandemic year compared with the pre-pandemic year (z=3.63, P<0.001). During the pre-pandemic year, the main reasons for temporary donor deferral were inappropriate hemoglobin levels, inappropriate blood pressure, heart rate and/or rhythm, recent tattoos and/or body piercing, and recent surgery and/or intervention. Despite the number of deferrals due to inappropriate hemoglobin levels during the first pandemic year decreasing by 20.31 percent, the proportion of temporary deferrals significantly increased (z=7.72, P<0.001) compared with the pre-pandemic year. A similar situation was observed when analyzing the deferrals due to inappropriate blood pressure, heart rate, and/or rhythm. During the first pandemic year, the number of deferrals decreased by 7.79 percent, but the proportion of such deferrals of all temporary deferrals significantly increased (z=6.35, P<0.001) compared to the pre-pandemic year. The number of deferrals due to recent tattoos and/or body piercing, as well as surgery and/or intervention, decreased by 42.41 and 36.54%, respectively, while there was a statistically significant decrease in the proportions of all temporary deferrals (z=4.35, P<0.001 and z=2.51, P<0.05, respectively). There were no statistically significant differences in the proportions of temporary deferrals due to inappropriate levels of hemoglobin, inappropriate blood pressure, heart rate and/or rhythm, and recent tattoos and/or body piercing between the third pandemic and pre-pandemic years. There was a 33.88% increase in temporary deferrals due to recent surgery and/or intervention between the third pandemic year and the pre-pandemic year. There was a statistically significantly higher proportion of deferrals due to this reason (z=8.1, P<0.001) during the third pandemic year compared to the pre-pandemic year.

**Table 3. Blood donors’ deferrals in Lithuania between April 2019 and March 2023**

| Dimensions | Years | | | | % change (+/-), comparing  Pre-pandemic year with | | |
| --- | --- | --- | --- | --- | --- | --- | --- |
|  | Pre-pandemic  No (%) | 1^st^ pandemic  No (%) | 2^nd^ pandemic  No (%) | 3^rd^ pandemic  No (%) | 1^st^ pandemic | 2^nd^  pandemic | 3^rd^  pandemic |
| Attempts to donate | 122509 | 108058 | 118890 | 124425 | -11.80 | -2.95 | +1.56 |
| Pre-donation deferrals | 17188 (14.03*) | 12649 (11.71*) | 15269 (12.83*) | 15428 (12.40*) | -26.41 | -11.16 | -10.24 |
| *Permanent deferrals* | | | | | | | |
| Positive TTI markers | 251 (57.44**) | 154 (55.40**) | 174 (51.33**) | 168 (44.68**) | -38.65 | -30.68 | -33.07 |
| Somatic illnesses or conditions | 156 (35.70**) | 87 (31.29**) | 127 (37.46**) | 154 (40.96**) | -44.23 | -18.59 | -1.28 |
| Infectious diseases | 6 (1.37**) | 4 (1.44**) | 0 (0.0**) | 12 (3.19**) | -33.33 | N/A | +100.0 |
| Drug usage | 2 (0.46**) | 1 (0.36**) | 4 (1.18**) | 3 (0.80**) | -50.0 | +100.0 | -25.0 |
| Other reasons | 22 (5.03**) | 32 (11.51**) | 34 (10.03**) | 39 (10.37**) | +45.45 | +54.55 | +77.27 |
| All | 437 | 278 | 339 | 376 | -36.38 | -22.43 | -13.96 |
| *Temporary deferrals* | | | | | | | |
| Inappropriate level of hemoglobin | 9547 (56.99**) | 7608 (61.50**) | 9632 (64.51**) | 8722 (57.95**) | -20.31 | +0.89 | -8.64 |
| Inappropriate blood pressure, heart rate, or rhythm | 1528 (9.12**) | 1409 (11.39**) | 1373 (9.20**) | 1410 (9.37**) | -7.79 | -10.14 | -7.72 |
| Surgery operation and/or intervention | 676 (4.04**) | 429 (3.47**) | 786 (5.26**) | 905 (6.01**) | -36.54 | +16.27 | +33.88 |
| Medication usage | 421 (2.51**) | 256 (2.07**) | 300 (2.01**) | 486 (3.23**) | -39.19 | -28.74 | +15.44 |
| Tattoos and/or body piercing | 797 (4.76**) | 459 (3.71**) | 658 (4.41**) | 699 (4.64**) | -42.41 | -17.44 | -12.30 |
| Refusal to donate | 217 (1.30**) | 148 (1.20**) | 106 (0.71**) | 160 (1.06**) | -31.80 | -51.15 | -26.27 |
| Inappropriate body weight and/or age | 118 (0.70**) | 59 (0.48**) | 74 (0.50**) | 53 (0.35**) | -50.0 | -37.29 | -55.08 |
| Other reasons | 3447 (20.58) | 2003 (16.19) | 2001 (13.40**) | 2617 (17.39**) | -41.89 | -41.95 | -24.08 |
| All | 16751 | 12371 | 14930 | 15052 | -26.15 | -10.87 | -10.14 |

*Deferral rate

**From total donations during particular year

Table 4 presents the prevalence of confirmed TTI disease markers in first-time and repeat/regular donations. There was a statistically significant (z=3.01, P<0.05) lower prevalence of HBV infection among first-time donors during the third pandemic year than during the pre-pandemic year. Moreover, A statistically significant (z=2.14, P<0.05) lower prevalence of all positive TTI markers among first-time donations was estimated during the third pandemic year compared with the pre-pandemic year. Finally, there was a significantly lower prevalence of all positive TTI markers among all (first-time and repeat/regular) donations compared with the pre-pandemic year in the first (z=4.8, P<0.001), second (z=4.36, P<0.001), and third pandemic years (z=4.72, P<0.001).

The odds ratio for confirmed TTI disease markers in all donors are shown in Table 5. During the first pandemic year, the odds for HBV, HCV, and syphilis, as well as for all TTI markers, were significantly lower than those in the pre-pandemic year. Moreover, the odds for HBV and HCV and all TTI markers during the second and third pandemic years were significantly lower than those in the pre-pandemic year.

**Table 4. The prevalence of confirmed transfusion-transmitted infectious disease markers in Lithuania blood donors between April 2019 and March 2023**

| Item | Pre-pandemic year | | 1^st^ pandemic year | | 2^nd^ pandemic year | | 3^rd^ pandemic year | |
| --- | --- | --- | --- | --- | --- | --- | --- | --- |
|  | First-time  № (%) | Repeat/regular  № (%) | First-time  № (%) | Repeat/regular  № (%) | First-time  № (%) | Repeat/regular  № (%) | First-time  № (%) | Repeat/regular  № (%) |
| Donations | 16820 | 88501 | 9368 | 86041 | 10949 | 92682 | 11756 | 97241 |
| *Confirmed TTI markers* | | | | | | | | |
| HBV | 69  (0.410) | 7  (0.008) | 29  (0.309) | 3  (0.003) | 33  (0.301) | 6  (0.006) | 24  (0.204) | 6  (0.006) |
| HCV | 53 (0.315) | 7  (0.008) | 23  (0.245) | 8  (0.009) | 24  (0.219) | 5  (0.005) | 30  (0.255) | 7  (0.007) |
| Syphilis | 31  (0.184) | 9  (0.010) | 16  (0.170) | 5  (0.006) | 22  (0.201) | 11  (0.012) | 26  (0.221) | 7  (0.007) |
| HIV 1 and 2 | 2  (0.012) | 3  (0.003) | 0  (0.0) | 5  (0.006) | 2  (0.018) | 2  (0.002) | 1  (0.009) | 5  (0.005) |
| All | 155 (0.921) | 26  (0.029) | 68  (0.726) | 21  (0.024) | 81  (0.739) | 24  (0.026) | 81  (0.689) | 25  (0.025) |

**Table 5. Odds ratio for confirmed transfusion-transmitted infectious disease markers for all donations in pre-pandemic year compared to pandemic years**

| Confirmed infection marker | 1^st^ pandemic year | | | 2^nd^ pandemic year | | | 3^rd^ pandemic year | | |
| --- | --- | --- | --- | --- | --- | --- | --- | --- | --- |
|  | Odds ratio | 95% CI | P-value | Odds ratio | 95% CI | P-value | Odds ratio | 95% CI | P-value |
| HBV | 0.47 | 0.31-0.70 | <0.001 | 0.52 | 0.35-077 | <0.001 | 0.38 | 0.25-0.58 | <0.001 |
| HCV | 0.57 | 0.37-0.88 | <0.05 | 0.49 | 0.32-0.77 | <0.05 | 0.48 | 0.31-0.75 | <0.001 |
| Syphilis | 0.57 | 0.34-0.98 | <0.05 | 0.84 | 0.53-1.33 | >0.05 | 0.80 | 0.50-1.26 | >0.05 |
| HIV 1 and 2 | 1.10 | 0.32-3.81 | >0.05 | 0.81 | 0.22-3.03 | >0.05 | 1.16 | 0.35-3.80 | >0.05 |
| All | 0.54 | 0.42-0.70 | <0.001 | 0.56 | 0.46-0.75 | <0.001 | 0.57 | 0.45-0.72 | <0.001 |

**Discussion**

To the best of our knowledge, this is the first study to describe the impact of the COVID-19 pandemic on blood donation and donor profiles over an extended period, specifically covering three years of the pandemic. This survey highlights the specific challenges faced during different years of the pandemic and indicates trends toward returning to the usual pre-pandemic situation.

During the first year of the pandemic, the number of blood donations and their components decreased by 9.41 percent in Lithuania. Whole blood donations decreased by 10.88 percent compared to the pre-pandemic years. The decrease in the number of donations was lower than that in other countries [6,17]; however, this could have been influenced by a different assessment period. When analyzing the location of blood and its component donations, it was observed that the number of donations during mobile blood drives was nearly one-third (31.8 percent) lower than that in the pre-pandemic years. Furthermore, a significant decrease in mobile blood drives was observed in other studies as well [8]. Notably, the proportion of first-time blood or component donations decreased by 44.30 percent in Lithuania during the first pandemic year, with similar trends during the second and third pandemic years. In contrast, studies in other countries showed increased first-time donations during the pandemic [7,8].

Analysis of blood and its component donations by donor age and sex showed that the largest decrease in the number of donations was observed in the group of donors aged < 25 years. The significant decrease in donations from the youngest donor group is probably in line with the reduction in first-time donations. A slight increase in the number of donations during the first pandemic year was observed only in men aged 35–44 years, 45–54 years, and 55–64 years. Generally, men were more active in donating blood and blood components during the first pandemic year, showing a small increase in proportion compared to the pre-pandemic year. Similar to this study, a significant decrease was seen in donors not more than 30 years old in the USA [8], while a study in Iran showed significantly greater donations of donors within the age group of 45-54 years in 2020 [11].

During the second year of the pandemic, donations of blood and its components in Lithuania nearly reached pre-pandemic levels, and in the third year, donations were 3.45 percent higher than in pre-pandemic years. The number of donations during mobile blood drives started to increase in the second and third years of the pandemic but remained at a lower level compared to the pre-pandemic year. When evaluating the decreasing trend in blood and its component donations during mobile blood drives over the pandemic period, the observed decrease did not seem to impact the overall number of donations.

Unexpectedly, the significant drop in donations in the group of donors under the age of 25 during the first pandemic year remained almost unchanged during the second and third years. The number of first-time donations showed an increasing trend during the second and third pandemic years, after a significant drop during the first pandemic year, but remained at a very low level (30.11 percent less during the third pandemic year compared to the pre-pandemic year). The proportion of first-time donations during the third pandemic year was significantly lower than that during the pre-pandemic year. These facts are of great concern because it is important to continuously maintain an adequate donor base with a sufficient proportion of young donors [18]. During the first year of the pandemic, donations from repeat/regular donors decreased by 2.78 percent compared to pre-pandemic years. However, during the second and third years of the pandemic, they increased (4.70 percent and 9.86%, respectively) compared to the pre-pandemic years. This confirms that repeat/regular blood and blood component donors are crucial during pandemics and other force majeure situations[19-22]. Nevertheless, the pool of repeat and regular donors must be supplemented with first-time donors.

During the first pandemic, the number of attempts to donate blood or its components decreased by 11.80 percent compared with the pre-pandemic year. The deferral rate also decreased (11.71 vs 14.03). During the third pandemic, the number of attempts to donate increased by 1.56 percent compared to the pre-pandemic year, while the deferral rate decreased. The increasing number and proportion of repeat and regular donations can probably explain this positive trend. Analyzing the trends of temporary and permanent deferrals, there was a decrease during the first pandemic year compared to the pre-pandemic year. However, the number of deferrals during the following pandemic years indicated a gradual approach toward pre-pandemic levels. Moreover, a decreased donor deferral rate during the COVID-19 pandemic was found in India [23], while a study in Iran showed opposite results [11].

The prevalence of all positive TTI markers among blood and its component donors significantly decreased during the pandemic years in Lithuania, and the estimated lower odds compared to the pre-pandemic year. First-time blood donors have a greater residual risk for positive TTI markers than repeat donors [24-26]; thus, a decrease in positive TTI markers during the pandemic can be explained by the fact that there was a decrease in donations of first-time blood and its components.

In this study, statistical data on blood and its component donations, as well as statistical information on blood donors, were analyzed. Therefore, a limitation of this study is that the demand for blood and its components was not assessed. Other studies have revealed that a reduction in donor numbers has largely been matched by a decreased demand for transfusion [27] and a reduction in hospital activities, especially elective surgery and strict blood patient management, which are thought to reduce the demand for blood transfusion [28]. This study did not analyze why donors did not donate blood or its components less frequently during the COVID-19 pandemic. A survey conducted in seven European countries (Denmark, France, Germany, Italy, Portugal, the Netherlands, and the UK) showed that approximately half of all donors reported that they donated less than they would normally, which suggests a concerning drop in blood donations throughout the COVID-19 period [29].

**Conclusions**

Most dimensions of blood and its component donations and donor characteristics have returned to pre-pandemic-year levels or even show positive trends. However, the concern is the decrease in donations from first-time and young donors under 25 years of age. Blood establishments need to review their strategies for recruiting new blood and blood component donors, with special attention paid to attracting young people.

Further studies are recommended to evaluate the motives of blood donors under 25 years of age and those who avoided donating during the COVID-19 pandemic.

**References**

1. World Health Organization. Coronavirus disease (COVID-19) pandemic [Cited June 20, 2023]. Available from: https://www.who.int/europe/emergencies/situations/covid-19

2. Alizadeh H, Sharifi A, Damanbagh S, Nazarnia H, Nazarnia M. Impacts of the COVID-19 pandemic on the social sphere and lessons for crisis management: a literature review. Nat Hazards (Dordr). 2023 Apr 10:1-26. doi: 10.1007/s11069-023-05959-2. Epub ahead of print. PMID: 37360799; PMCID: PMC10088618.

3. OECD (2023), Ready for the Next Crisis? Investing in Health System Resilience, OECD Health Policy Studies, OECD Publishing, Paris, <https://doi.org/10.1787/1e53cf80-en>.

4. Bates I, Owusu-Ofori S. Blood Transfusion. Manson's Tropical Diseases. 2009:229–34. doi: 10.1016/B978-1-4160-4470-3.50018-5. Epub 2020 Jun 22. PMCID: PMC7310926.

5. World Health Organization. (‎2023)‎. Guidance on ensuring a sufficient supply of safe blood and blood components during emergencies. World Health Organization. <https://iris.who.int/handle/10665/366870>. License: CC BY-NC-SA 3.0 IGO

6. Chiem C, Alghamdi K, Nguyen T, Han JH, Huo H, Jackson D. The Impact of COVID-19 on Blood Transfusion Services: A Systematic Review and Meta-Analysis. Transfus Med Hemother 2021;30:1-12. doi: 10.1159/000519245. PMID: 34934412; PMCID: PMC8678226.

7. Divkolaye N, Arabkhazaeli A, Hajibeigi B, Eshghi P. The impact of COVID-19 on blood safety and availability in the Islamic Republic of Iran. East Mediterr Health J 2022;28:823-828. doi: 10.26719/emhj.22.080. PMID: 36515446.

8. Gammon RR, Prichard AB, Gannett MS, Yordanov B. The effect of COVID-19 on blood donation habits. Transfusion 2021;61:1134-1140. doi: 10.1111/trf.16278. Epub 2021 Feb 10. PMID: 33565620; PMCID: PMC8014865.

9. Vassallo RR, Bravo MD, Kamel H. Pandemic blood donor demographics - Do changes impact blood safety? Transfusion 2021;61:1389-1393. doi: 10.1111/trf.16320. Epub 2021 Feb 20. PMID: 33554333; PMCID: PMC8013315.

10. Cardona-Arias JA, Higuita-Gutiérrez LF. Impact of COVID-19 Pandemic on Seroprevalence of HIV, HBV, HCV and HTLV I-II in a Blood Bank in Medellín, Colombia, 2019-2022. Trop Med Infect Dis 2023;8:118. doi: 10.3390/tropicalmed8020118. PMID: 36828534; PMCID: PMC9964071.

11. Rafiee MH, Kafiabad SA, Maghsudlu M. Analysis of blood donors' characteristics and deferrals related to COVID-19 in Iran. Transfus Apher Sci 2021;60:103049. doi: 10.1016/j.transci.2020.103049. Epub 2021 Jan 2. PMID: 33468406; PMCID: PMC7833597.

12. Lithuanian Institute of Hygiene. Blood donor and donation statistics from Q2 2019 to Q1 2023, Blood Donor’s Registry data (in Lithuanian). [Cited May 11, 2023]. Available from: https://hi.lt/en/blood-donor-register.html

13. Decision of the Government of the Republic of Lithuania on 14-03-2020 № 270, declaring a quarantine in the territory of the Republic of Lithuania (in Lithuanian). [Cited June 25, 2023]. Available from: <https://e-seimas.lrs.lt/portal/legalAct/lt/TAD/deaf8694663011eaa02cacf2a861120c>

14. Decision of the Governement of the Republic of Lithuania on 10-06-2020 № 579, canceling the Decision of the Government of the Republic of Lithuania on 14-03-2020 № 270 (in Lithuanian). [Cited October 2, 2023]. Available from: https://e-seimas.lrs.lt/portal/legalAct/lt/TAD/99f1d6b6abcd11ea8aadde924aa85003?jfwid=c2vttcb6e

15. Ministry of Health of the Republic of Lithuania. Order of Minister of Health on 13-10-2014 № V-1060, adopting procedure on blood donors’ testing for infectious disease markers (in Lithuanian). [Cited June 25, 2023]. Available from: https://e-seimas.lrs.lt/portal/legalAct/lt/TAD/792703e0589c11e49df480952cc07606?jfwid

16. Ministry of Health of the Republic of Lithuania. Order of Minister of Health on 04-02-2005 № V-84, adopting procedure on check of blood donors’ health status and donation procedure (in Lithuanian). [Cited June 25, 2023]. Available from: https://e-seimas.lrs.lt/portal/legalAct/lt/TAD/TAIS.249902/asr

17. García-Erce JA, Romón-Alonso Í, Jericó C, Domingo-Morera JM, Arroyo-Rodríguez JL, Sola-Lapeña C, et al. Blood Donations and Transfusions during the COVID-19 Pandemic in Spain: Impact According to Autonomous Communities and Hospitals. Int J Environ Res Public Health 2021;18:3480. doi: 10.3390/ijerph18073480. PMID: 33801650; PMCID: PMC8037479.

18. Carter MC, Wilson J, Redpath GS, Hayes P, Mitchell C. Donor recruitment in the 21st century: Challenges and lessons learned in the first decade. Transfus Apher Sci. 2011;45:31-43. doi: 10.1016/j.transci.2011.06.001. Epub 2011 Jul 26. PMID: 21795117.

19. McGann PT, Weyand AC. Lessons learned from the COVID-19 pandemic blood supply crisis. J Hosp Med 2022 ;17:574-576. doi: 10.1002/jhm.12843. Epub 2022 Jun 21. PMID: 35729854; PMCID: PMC9349403.

20. Chegini A. Evaluating the Importance of Patient Blood Management During COVID-19 Pandemic. Anesth Pain Med 2022;11:e112910. doi: 10.5812/aapm.112910. PMID: 35291403; PMCID: PMC8909534.

21. World Health Organization. (‎2020)‎. Maintaining a safe and adequate blood supply during the pandemic outbreak of coronavirus disease (‎COVID-19)‎: interim guidance, 20 March 2020. World Health Organization. <https://iris.who.int/handle/10665/331523>. License: CC BY-NC-SA 3.0 IGO

22. Interim guidance on maintaining a safe and adequate blood supply and collecting convalescent plasma in the context of the COVID-19 pandemic. World Health Organization 2021. Available from: https://www.who.int/publications/i/item/WHO-2019-nCoV-BloodSupply-2021-1

23. Routray SS, Ray GK, Prakash S, Sahu A, Naik A, Mukherjee S. Impact of COVID-19 on blood donor deferral patterns during the COVID-19 pandemic: A retrospective analysis. Vox Sang 2022;117:656-663. doi: 10.1111/vox.13240. Epub 2022 Feb 3. PMID: 35114023.

24. Velati C, Romanò L, Piccinini V, Marano G, Catalano L, Pupella S, et al. Prevalence, incidence and residual risk of transfusion-transmitted hepatitis C virus and human immunodeficiency virus after the implementation of nucleic acid testing in Italy: a 7-year (2009-2015) survey. Blood Transfus 2018;16:422-432. doi: 10.2450/2018.0069-18. Epub 2018 Jun 26. PMID: 30036178; PMCID: PMC6125236.

25. Velati C, Romanò L, Pati I, Marano G, Piccinini V, Catalano L, et al. Prevalence, incidence and residual risk of transfusion-transmitted hepatitis B virus infection in Italy from 2009 to 2018. Blood Transfus 2019;17:409-417. doi: 10.2450/2019.0245-19. . Epub 2019 Dec 11. PMID: 31846606; PMCID: PMC6917538.

26. O'Brien SF, Yi QL, Fan W, Scalia V, Goldman M, Fearon MA. Residual risk of HIV, HCV and HBV in Canada. Transfus Apher Sci 2017;56:389-391. doi: 10.1016/j.transci.2017.03.010. Epub 2017 Mar 18. PMID: 28389206.

27. Stanworth SJ, New HV, Apelseth TO, Brunskill S, Cardigan R, Doree C, et al. Effects of the COVID-19 pandemic on supply and use of blood for transfusion. Lancet Haematol 2020;7:e756-e764. doi: 10.1016/S2352-3026(20)30186-1. Epub 2020 Jul 3. PMID: 32628911; PMCID: PMC7333996.

28. Gehrie EA, Frank SM, Goobie SM. Balancing Supply and Demand for Blood during the COVID-19 Pandemic. Anesthesiology 2020;133:16-18. doi: 10.1097/ALN.0000000000003341. PMID: 32550680; PMCID: PMC7176270.

29. Chandler T, Neumann-Böhme S, Sabat I, Barros PP, Brouwer W, van Exel J, et al. Blood donation in times of crisis: Early insight into the impact of COVID-19 on blood donors and their motivation to donate across European countries. Vox Sang 2021;116:1031-1041. doi: 10.1111/vox.13103. Epub 2021 Apr 9. PMID: 33835509; PMCID: PMC8250750.
