# Supplementary material for: Blood donations and donors’ profile in Lithuania: Trends for coming back after the COVID-19 outbreak
Source: PLoS One. 2024 Jan 25;19(1):e0297580. doi: 10.1371/journal.pone.0297580 (PMC10810517; doi:10.1371/journal.pone.0297580)
Supplement: Attachment — Submitted filename: Response to Reviewers.docx [file pone.0297580.s009.docx]

Editors

PLOS ONE

December 4, 2023

Dear Editor,

We thank you and the reviewers for a thorough reading and constructive comments to improve our manuscript and for the opportunity to revise and resubmit it. We are pleased to submit the improved research article “Blood donations and donors' profile in Lithuania: trends for coming back after the COVID-19 outbreak?“ for your consideration for publication in PLOS ONE. Below you will find our responses to Reviewers.

On behalf of the authors, I thank you for your consideration of this resubmission. We appreciate your time and look forward to your response.

Sincerely,

Prof. Vytenis Kalibatas, MD, MPH, PhD (corresponding author)

Department of Health Management,

Lithuanian University of Health Sciences

Tilžės str. 18, LT-47181, Kaunas, Lithuania

E-mail: vytenis.kalibatas@lsmuni.lt

Responses to Reviewers:

| **№** | **Comments** | **Responses** |
| --- | --- | --- |
| Reviewer #1 | | |
| 1. | In Statistical analysis it should be written as IBM SPSS software (version 29) instead of SPSS | Accepted.  We added the missed word in the sentence. Thank you. |
| 2. | In page no 13(146,147,148,149), this analysis is not mentioned anywhere | Done.  We added an additional information on remunerated and voluntary non-remunerated donations in the Table 1. |
| 3. | In each 'Table' z score and p-value should be included | Done.  We added an additional information on the presence of statistically significant differences while compared proportions between each other years in each table, and included 4 additional supplement tables with the score values. |
| 4. | Operational definition of 'regular donor' should be clarified | Done.  We provided the definitions of first-time, repeat and regular donor in Donor screening and deferral section. |
| 5. | In Table 5 pre-pandemic year Odds ratio, 95% CI, p-value not mentioned | The odds ratio is the set of two odds. In our case – the odds of confirmed TTI in pre-pandemic year versus the odds of confirmed TTI in the first pandemic (second pandemic, third pandemic) year. For example, the odds ratio 0.47 for HBV in the first-pandemic year (Table 5) means that there is a 47% decrease in the odds of HBV during first pandemic year, compared with the pre-pandemic year. Thus, there is no point in calculating and mentioning the odds ratio for the same year (pre-pandemic versus pre-pandemic) since the odds ratio and P values will be the same, i.e., equal to 1.00.  Thank you for all remarks! |
| Reviewer #2 | | |
| 1. | Line 121 (Statistical analyses): Authors should consider adding extra bit of information to bring clarity to the assumptions for the data analyses. From the results and tables presented in the manuscript, the authors used the pre-pandemic blood donation stocks as the baseline to which the pandemic years (1, 2, 3) were compared to. Please consider making this explicit under the statistical analyses section. | Considering all your comments, we have opted to include statistical information for comparisons not only between the pre-pandemic year and all the pandemic years but also among the different pandemic years. Otherwise, we would have done the suggested corrections in the Statistical analysis section.  Thank you. |
| 2. | Line 121 (statistical analyses) - Is there any reason why the pandemic years were not compared to each other? as in multiple comparisons? How does the reader judge whether the changes observed in pandemic year 1 significantly differed from pandemic year 2 or 3? This might become important when discussing as to whether specific public health policies implemented in Lithuania during the pandemic had significant change in public perception to blood donation for example. | Done.  We fully acknowledge the significance of this issue and, in response to your suggestion, have included additional information on comparisons among the different pandemic years. Initially, our intent was to avoid overloading the manuscript with statistical numbers; however, your insightful comments have led us to include this important information (refer to Tables 1-4 and the new Table 6, along with supplementary details in the Results section).  We sincerely appreciate your valuable remark. Thank you very much for bringing this to our attention. |
| 3. | The descriptions of Table 1 in Line 162 - 169 should precede Table 2, but not come after Table 2. | Done. |
| 4. | The statement "During the pandemic, the number of pre-donation deferrals decreased (Table 3)" in line 190 - 191 does not seem to be a complete statement. | Done.  We corrected the sentence as follows: “During the pandemic, the number of pre-donation deferrals decreased comparing with the pre-pandemic year” (line 248-249).  Thank you. |
| 5. | The descriptions to table line 228 - 232 should come after table 4. | Done. |
| 6. | The discussion could be improved if the authors critically engage the data, particularly to highlight plausible reasons as to why there were differences in the data reported herein compared to studies from other places. For example, in line 261-262, the authors references studies that gave contrasting findings, whereas in line in line 258-259 (and line 303), the authors cite studies that gave similar results. What might have accounted for these differences in the reported studies? For example, were there differences in easing of restrictions in Lithuania compared to other countries? Even in this data being reported, what were plausible explanations for the increasing trend towards donations after the dip in the first pandemic year? Could this be attributed to public health campaigns? What led to improved public perceptions to blood donation in Lithuania? What might have the blood collection centers in Lithuania done differently during the course of the pandemic to have led to improved blood donation trend. | Done.  We acknowledge the importance of the issues you have raised. In response to your insightful comments, we have made significant additions to the Discussion section. Specifically, we have detailed the actions taken during the pandemic in Lithuania, which not only shed light on crucial aspects of our findings but also facilitate comparisons with actions taken in other countries.  We greatly appreciate your valuable remarks. |
| 7. | Again, why was the blood donation trends in the <25 years (in this study) very different from the other age groups? | Regrettably, we cannot directly address this crucial question. However, we can offer some general reflections, such as the closure and transition to virtual platforms of educational facilities, along with the decline of activities in open public places where mobile sessions were previously conducted. Additionally, we posit that reinforced attention to regular and repeat donors coupled with diminished recruitment of new donors could, at least in part, explain the decline in the number of first-time donors (including those under 25 years old), and donations in Lithuania (please see the Discussion section). Therefore, we recommended further studies on evaluation of the motives of blood donors under 25 years of age and those who avoided donating during the COVID-19 pandemic (please see Conclusions).  Thank you! |
| Reviewer #3 | | |
| 1. | The section of ethical consideration needs a minor revision. | Accepted.  We added the proposed sentence at the beginning of Ethical consideration section. Thank you. |
| 2. | Titles of the tables need revision and suggestions have been given. | Accepted.  We reformulated the titles of the tables according to the suggestions. Thank you. |
| 3. | Authors should include a "trend" graph in addition to the table 1. This will display the subject under consideration more appropriately. | Done.  We included Figure 1, which shows the total number of blood and blood components’ donations, the number of first-time and repeat/regular donations during pre-pandemic and pandemic years.  Thank you! |
| Reviewer #4 | | |
| 1. | I think the paper would be better reformatted as a short scientific report rather than a full length article to have a better chance of acceptance | Thank you for your insightful comments. We appreciate your observation. The decision to categorize our manuscript as a full-length article was based on the consideration of numerous indicators and their values spanning a four-year period. Our intention was to present comprehensively these indicators to facilitate a thorough understanding and evaluation of the overall situation. We acknowledge the significance of your suggestion and we will incorporate it into consideration while preparing the new manuscript. |
| 2. | I also have a specific recommendation to better interpret the important finding of this study regarding the observed lower prevalence of transfusion-transmitted infectious markers (TTI) cases among donations compared to the year before the pandemic. I may somewhat be agree with the respected authors that this can be due to a decrease in the number of first-time blood donor during pandemic, but according to my years of experience, there are always some donors who sometimes want to donate blood to monitor their health status, some of whom have been exposed to high-risk sexual behaviors. However, such people may be less motivated to donate in difficult pandemic conditions than regular donors and therefore it is very likely that the number of such donors has decreased significantly during the COVID-19 pandemic, which is reflected in the decrease in positive cases. | Done.  We acknowledge the significance of addressing the issue of test-seeking donors in the context of the decline in Transfusion-Transmissible Infections (TTI) among blood and its components donors during the pandemic. We could not find any publication on the behavior and motivation of test-seeking blood donors specifically during the COVID-19 pandemic. Nevertheless, we have incorporated relevant information in the Discussion section (lines 412-416) when deliberating on the observed decrease in TTI among donors during the pandemic.  We appreciate your astute observation and the importance of this aspect, and we thank you for bringing it to our attention. |
| 3. | On the other hand, the reduction of high-risk sexual behaviors during the pandemic can also be another reason for this finding. | Done.  We appreciate and agree with your observation regarding the decline of TTI among blood donors during the pandemic, suggesting a possible link to a reduction in high-risk behavior. Despite an exhaustive literature search, we were unable to find supporting information for this particular assertion. Conversely, our investigation did reveal information indicating an increase in high-risk behavior among certain demographic groups during the pandemic, although not specifically among blood donors. Nevertheless, we have addressed this aspect in the Discussion section (lines 416-419).  Thank you! |
