# Supplementary material for: Blood donations and donors’ profile in Lithuania: Trends for coming back after the COVID-19 outbreak
Source: PLoS One. 2024 Jan 25;19(1):e0297580. doi: 10.1371/journal.pone.0297580 (PMC10810517; doi:10.1371/journal.pone.0297580)
Supplement: Attachment — Submitted filename: Response to Academic Editor.docx [file pone.0297580.s010.docx]

Dr Enoch Aninagyei
Academic Editor
PLOS ONE

December 22, 2023

Dear Dr Enoch Aninagyei,

We thank you for a thorough reading and constructive comments to improve our manuscript and for the opportunity to revise and resubmit it. We are pleased to submit the improved research article “Blood donations and donors' profile in Lithuania: trends for coming back after the COVID-19 outbreak“ for your consideration for publication in PLOS ONE. Below you will find our responses to Academic editor comments.

On behalf of the authors, I thank you for your consideration of this resubmission. We appreciate your time and look forward to your response.

Sincerely,

Prof. Vytenis Kalibatas, MD, MPH, PhD (corresponding author)

Department of Health Management,

Lithuanian University of Health Sciences

Tilžės str. 18, LT-47181, Kaunas, Lithuania

E-mail: vytenis.kalibatas@lsmuni.lt

Responses to Academic editor:

| **№** | **Comments** | **Responses** |
| --- | --- | --- |
| 1. | Title:  Considering rephrasing not in a question form | Done |
| 2. | Abstract:  Line 35: Indicate the full forms of all abbreviations on first mention | Done |
| 3. | Abstract:  Lines 31/39: under 25 what? Years? | Corrected in the Abstract and other sections of the manuscript |
| 4. | Methods  Line 89: Indicate the date the restrictions were eased | Done |
| 5. | Methods  Lines 87-95: The pandemic was declared over, can you add the post-pandemic period as well | We can theoretically assume that the beginning of the post-pandemic period starts after May 5, 2023. On May 5, 2023, the WHO Emergency Committee on COVID-19 recommended recognizing that, given that the disease was well-established and ongoing, it no longer fit the definition of a Public Health Emergency of International Concern (PHEIC). Unfortunately, we could not find any official statement or scientific publication on the earlier beginning of “post-pandemic” period, which could cover the period of our study. Our study covered the period from April 1, 2019 until March 31, 2023, so we did not analyze the data in the post-pandemic period (according to WHO statement on May 5, 2023). |
| 6. | Methods  Line 108: italicise Treponema Pallidum | Done |
| 7. | Methods  Line 134: Indicate ethics approval number | The Kaunas Regional Biomedical Research Ethics Committee did not issue an ethics approval number, since this survey is not the subject of bioethical regulation (because generalized, fully anonymized, and publicly available data have been used). As outlined in the Ethical considerations section, “The Kaunas Regional Biomedical Research Ethics Committee reviewed the methodology of this survey and deemed the investigation an evaluation of service, not requiring review by an ethics committee, as the direct object of this study was not a specific person or person´s health". Due to misinterpretation, we removed the first sentence in the Ethical consideration section. |
| 8. | Results  Table 1 and subsequent tables, can you segregate data to include post-pandemic period? | We acknowledge the concern regarding the post-pandemic period, and we were ready to segregate and re-calculate data in order to include the "post-pandemic" period. But, as previously outlined, we could not find any other official statement (except the WHO statement on May 5, 2023) or scientific publication on the beginning of “post-pandemic” period, which could cover the period of our study. Nevertheless, the primary focus of our study was to assess the number, types, and safety of blood and its components' donations, along with the demographic profile of blood donors. Despite how we name a year (or shorter period) after the COVID-19 outbreak, our approach involved a comparison of each pre-pandemic year with subsequent years of post-COVID-19 outbreak, ensuring statistically reliable comparisons across equivalent time periods. |

Thank you very much for all remarks and comments!
