# Supplementary material for: Blood donations and donors’ profile in Lithuania: Trends for coming back after the COVID-19 outbreak
Source: PLoS One. 2024 Jan 25;19(1):e0297580. doi: 10.1371/journal.pone.0297580 (PMC10810517; doi:10.1371/journal.pone.0297580)
Supplement: Attachment — Submitted filename: Reviewer comment for Manuscript Number PONE-D-23-28681R2.docx [file pone.0297580.s007.docx]

SUMMARY

This is an important manuscript with relevant public health implications that could assist public health experts to plan during pandemic situations to ensure sustainable blood stocks. The manuscript is well written. However, the discussion lacks a much needed critical engagement of the data in the light of other studies to provide a context for the data. The authors should consider addressing issues raised below to improve the manuscript.

Materials and methods

1. Line 121 (Statistical analyses): Authors should consider adding extra bit of information to bring clarity to the assumptions for the data analyses. From the results and tables presented in the manuscript, the authors used the pre-pandemic blood donation stocks as the baseline to which the pandemic years (1, 2, 3) were compared to. Please consider making this explicit under the statistical analyses section.

2. Line 121 (statistical analyses) - Is there any reason why the pandemic years were not compared to each other? as in multiple comparisons? How does the reader judge whether the changes observed in pandemic year 1 significantly differed from pandemic year 2 or 3? This might become important when discussing as to whether specific public health policies implemented in Lithuania during the pandemic had significant change in public perception to blood donation for example.

RESULTS

3.The descriptions of Table 1 in Line 162 - 169 should precede Table 2, but not come after Table 2.

4. The statement "During the pandemic, the number of pre-donation deferrals decreased (Table 3)" in line 190 - 191 does not seem to be a complete statement.

5. The descriptions to table line 228 - 232 should come after table 4.

DISCUSSION

1. The discussion could be improved if the authors critically engage the data, particularly to highlight plausible reasons as to why there were differences in the data reported herein compared to studies from other places. For example, in line 261-262, the authors references studies that gave contrasting findings, whereas in line in line 258-259 (and line 303), the authors cite studies that gave similar results. What might have accounted for these differences in the reported studies? For example, were there differences in easing of restrictions in Lithuania compared to other countries? Even in this data being reported, what were plausible explanations for the increasing trend towards donations after the dip in the first pandemic year? Could this be attributed to public health campaigns? What led to improved public perceptions to blood donation in Lithuania? What might have the blood collection centers in Lithuania done differently during the course of the pandemic to have led to improved blood donation trend.

2. Again, why was the blood donation trends in the <25 years (in this study) very different from the other age groups?

Since no one could predict when another pandemic might strike, critical exploration of the data that provides enabling context will enable public health experts in other countries to tap into this resource to ensure sustained blood availability of blood in the event of another pandemic.
